# Understanding Resilience Dimensions and Adaptive Strategies to the Impact of Recurrent Droughts in Borana Zone, Oromia Region, Ethiopia: A Grounded Theory Approach

**DOI:** 10.3390/ijerph14020118

**Published:** 2017-01-26

**Authors:** Zewdie Birhanu, Argaw Ambelu, Negalign Berhanu, Abraraw Tesfaye, Kifle Woldemichael

**Affiliations:** 1 Department of Health Education and Behavioral Sciences, Faculty of Public Health, Jimma University, Jimma 378, Ethiopia; 2 Department of Environmental Health Sciences and Technology, Faculty of Public Health, Jimma University, Jimma 378, Ethiopia; Argaw.ambelu@ju.edu.et; 3 Department of Health Economics, Management and Policy, Faculty of Public Health, Jimma University, Jimma 378, Ethiopia; negalign.berhanu@ju.edu.et; 4 Resilient Africa Network, Horn of Africa Resilience Innovation Lab, Jimma University, Jimma 378, Ethiopia; abrarawt@yahoo.com; 5 Department of Epidemiology, Faculty of Public Health, Jimma University, Jimma 378, Ethiopia; kifle.woldemichael@ju.edu.et

**Keywords:** resilience, recurrent droughts, disasters, shocks, adaptive capacity, coping strategies, pastoralists, Borana, Ethiopia

## Abstract

Recurrent shocks and stresses are increasingly deteriorating pastoralist communities’ resilience capacities in many aspects. A context specific resilience framework is essential to strengthen pastoralist community’s resilience capacity towards the impact of recurrent drought. Hence, the present study was aimed to develop a context specific and data driven resilience building framework towards impacts of recurrent droughts in the case of Borana pastoralists in Ethiopia. Qualitative grounded theory approach was employed to guide the study process. The data were collected through focus group discussions and in-depth interviews in two drought affected districts of Borana Zone during October 2013. The analysis was assisted by ATLAS. ti 7.1.4. The analysis provided a context specific resilience building conceptual tool, which consists of, closely interconnected, eight dimensions operating at multiple capacities and levels: environment (underlying vulnerability factor); livestock, infrastructures/social services, and wealth (immediate causes and effects); community network/social capital, as well as governance, peace and security (support and enabling factors oriented), psychosocial, and human capital (as eventual outcomes and impacts). The resilience capacities of these pastoralist communities have been eroded, leaving them without sufficient and effective adaptive strategies. The emergent resilience framework can serve as a useful guidance to design context-specific interventions that makes the people and the system resilient to the impacts of recurrent droughts.

## 1. Introduction

Disasters and other emergencies are increasingly becoming important health, socio-economic and development challenges all over the world. Over the past few decades, the burden of natural and human-made disasters has increased [1]. Human factors are increasingly underlying most disaster situations, contributing to and or aggravating either the causes or the effects of the recurrent disasters [2]. Most disasters often involve sudden shocks that disrupt the livelihoods of communities, infrastructure and institutions [2]. Even without sudden shocks, communities affected by some disasters such as drought face slow-onset and persistent stress that affects their wellbeing. The Millennium Development Goals (MDGs) and recently, Sustainable Development Goals (SDGs) are extremely challenged in many communities and countries by losses from disasters [3,4,5]. The 2030 Agenda for SDGs recognizes and reaffirms the urgent need to reduce the risk of disasters by reducing exposure and vulnerability of communities through building resilient infrastructures and system [4]. There are 25 targets related to disaster risk reduction in SDGs, proving the role of disaster risk reduction as a core development strategy and a cross cutting issue in development agendas [4]. Thus, building resilience and reducing disaster risk can significantly contribute to achievement of international development goals [4,5].

Drought is one of the most challenging shocks, affecting the lives of millions, the highest burden being among African nations [3]. Drought is generally a situation where there has not been enough rainfall over an extended period of time, usually for a season or more, leading to water shortages and producing a serious hydrologic imbalance [6]. Given the changing global climatic conditions, there is an increasing concern that droughts may be increasing in frequency and severity in the coming years [6]. Even though drought could affect any environment and communities, pastoralist communities are the most vulnerable ones that most frequently affected by dreadful drought [7].

In Ethiopia, pastoralist and agro-pastoralist communities represent 12% of the total population [8] and they are herding their livestock in the arid and semi-arid lowland areas of the country which are basically prone to rainfall variability and extreme drought [9]. They are endowed with 22% of the country’s cattle population and contribute 12%–16% of Ethiopia’s Gross Domestic Product (GDP) and 30%–35% of the agricultural GDP [6]. Despite their crucial contributions, they often suffer from frequent attacks of recurrent droughts.

Despite tremendous efforts to build the resilience capacities of Ethiopian pastoralists [10,11,12,13], they remained less resilient to the impacts of recurrent droughts. In order to improve effective community resilience capacities towards recurrent droughts or any other shocks and stresses, it is necessary to properly understand and conceptualize the concept of resilience [1]. Resilience is often defined and understood in different ways though many of these definitions have no significant conceptual differences. Many definitions often combine comparable key elements such as ability, mitigation, adaptation, coping, recovery, shocks, stresses, risks, withstand, resist, and vulnerability [14,15]. For simplicity and clarity, resilience can be understood as the capacity to withstand or absorb sudden or chronic shock; cope with temporary disruption while minimizing the damages and costs from hazard; restore after an event; manage or maintain basic functions and structures to become suitable for future situation [16]. For a community, in order to be fully resilient, resilience building efforts must be considered at multi-level, involving individuals, households, communities, and system [17]. People and system resilience is best built through thorough understanding of context specific resilience challenges and dimensions, vulnerability factors and adaptive and coping strategies [1,18]. Thus, the first step towards more concerted and coordinated global and local actions about disaster risk reduction must be a clear understanding of the local vulnerability factors and basing the interventions on contextually relevant framework [1]. In recent years, there is a growing understanding and advocacy for an innovative approach to resilience building efforts. For instance, Resilient African Network (RAN) assumed that “the resilience of people and systems will be strengthened by leveraging the knowledge, scholarship and creativity to incubate, test, and scale innovations that target capabilities and reduce vulnerabilities identified by a scientific, data-driven, and evidence-based resilience framework” [18]. This process requires analyzing the context and understanding resilience dimensions which guide the development of effective and innovative solutions which can improve overall people’s and system’s resilience capacities. Past scientific inquiries and resilience strengthening efforts mostly ignored this unique and critical step in the process of building or strengthening people’s and system’s resilience to impacts of shocks including recurrent droughts as most disaster risk reduction and response activities are often driven by top down approach [19,20,21,22]. Therefore, it is a critical juncture for resilience stakeholders to support and focus on understanding and building resilience in a specific context. As such, there is no universal resilience building strategies that works in every setting and contexts, affirming the need to develop a context specific resilience building interventions. We argue that there is no metrics used to quantify resilience; resilience and dimensions could differ across settings and will need to be carefully developed, tailored to the specific local context and constellation of dimensions in a particular community. Therefore, this qualitative study aimed at understanding the resilience vulnerability factors, adaptive and coping strategies to recurrent droughts as a basis for informing the development of data driven and context specific resilience dimensions which provide potential resilience intervention pathways among pastoralist communities of Borana Zone, Oromia Region, Ethiopia.

## 2. Materials and Methods

### 2.1. Study Setting 

The study has focused on two districts of Borana Zone (i.e., Dhas and Arero), Oromia Region, Southern Ethiopia. Borana Zone is one of the areas frequently and severely affected by recurrent droughts. The zone is situated between 3°36’–6°38’ North latitude and 3°43’–39°30’ East longitude. The altitude of the zone ranges between 1000 m to 1500 m above sea level. Ecologically, 70% of the zone landmass is classified as semi-arid lowland and has two rainy seasons of different duration. The main rainy season, locally called *Genna* runs from mid-March to May and the short rainy season called *Hageya* extends from September to mid-November. The semi-arid lowlands are predominantly flat, covered with bushes and shrubs [10,23,24]. As a result of recurrent droughts, the zone has frequently experienced immense loss of livestock, assets and human life for the last 50 years [11,25,26] with greater frequency in recent years than ever before. The zone and the study districts were chosen taking into account the frequency and severity of droughts. Arero district covers an area of 10,890 km^2^ and hosts a total population of about 50,000 of which 85% were pastoralists. According to the data obtained from the district, only 20% of the population had access to potable water sources. On the other hand, Dhas district stretches over an area of 3447 km^2^ with a total population of about 56,837 of which 87% were pastoralists. In this district, only 15% of the population had access to potable water sources [10]. Fieldwork was carried out from September to October 2013.

### 2.2. Study Design

Qualitative grounded theory approach was employed to guide the study process. Grounded theory is a qualitative research approach used to inductively develop dimensions that are grounded in systematically gathered and analyzed data. It starts with individual experiences and develops progressively more abstract conceptual categories to synthesize, explain, and understand data and to identify patterned relationships within it [27]. Thus, given the inherent aim of the current study (i.e., to identify resilience dimensions that are grounded), grounded theory approach was chosen to guide the study process.

### 2.3. Population and Sample

The data were collected through Focus Group Discussions (FGDs) and Key Informant Interviews (KIIs) from diverse community groups. The FGDs were conducted in four pastoral *Gandas* (the smallest administrative units in Oromia), i.e., two *Gandas* from each district. These *Gandas* were identified taking into account the extent of vulnerability to recurrent droughts. In each *Ganda*, a total of three FGDs were conducted, involving adult males, adult females and informal community leaders, making a total of twelve FGDs. Each FGD had eight to twelve participants who were purposively selected considering their age and experiences in the area. With respect to key informant interviews, in order to obtain deep and varied data regarding the phenomenon under consideration, thirty-six KIIs were conducted with various key informants at different levels, ranging from community level to federal level, involving concerned government sectors, Non-Governmental Organizations (NGOs), and a UN Agency. These key informants were purposively selected considering their experiences and expertise with respect to the phenomenon, looking into their direct or indirect involvement in the provision of supports to pastoralist communities in the context of recurrent droughts.

### 2.4. Data Collection Tools and Procedures

FGDs and interview guides were developed through intensive review of resilience literatures. The tool was organized under four major themes which included: (1) major shocks and stresses affecting the study communities; (2) vulnerability factors; (3) drivers of vulnerability; and (4) adaptive factors. Each of these major themes was accompanied by several possible probes and follow-up questions to get deep insight of community’s lived experiences with respect to recurrent drought. The tool was further refined by inputs from participants of a workshop that was held in Jimma, Ethiopia and pre-tested in order to ensure its relevance and appropriateness. Data collectors and supervisors were trained on conducting FGDs and qualitative interviews. The trainees comprised faculty and graduate students of Jimma University from diverse fields of study with appropriate technical mix. Each FGD was conducted at least by two individuals who served as moderator and note taker. All FGDs and KIIs were recorded using digital voice recorder.

### 2.5. Data Analysis

FGD and KII data were transcribed verbatim and then translated into English by language experts. The coding and further analysis of the data was assisted by ATLAS.ti 7.1.4 (Scientific Software Development GmbH, Berlin, Germany). The transcripts were read and re-read by research team who then assigned codes and came up with an initial coding structure. The initial codes were subjected to discussion and refinement throughout the data coding period as new codes emerged from the text. Then, codes were grouped into code families and sub-themes. Sub-themes were reviewed further to develop overarching themes (typologies) and typologies were the bases for deriving dimensions in the resilience assessment framework for the target communities. Code co-occurrences and code-primary document table outputs were applied to devise resilience dimensions and compute conceptual overlaps and linkages. The frequency of co-occurrence codes in each emerged theme and its strength of relationship was shown using co-occurrence coefficient (C-coefficient) value.

### 2.6. Definition of Terms

Concepts used in the article are defined as follows:

- Resilience: the capacity of people and systems to mitigate, adapt to and recover and learn from recurrent droughts in a manner that reduces vulnerability and increases wellbeing [18].
- Adaptive strategies are defined as those methods (e.g., Cattle segregation, enclosing grazing land) used by communities to manage the impacts of recurrent droughts and have positive effects on community’s and system resilience to reduce long term vulnerability and improve wellbeing.
- Coping strategies are short term reactions to the effects and impacts of droughts (e.g., Charcoal production) which actually do not improve community and system resilience; they rather erode or do not lead to net improvement in resilience.
- Shock: the occurrence of recurrent droughts resulting in a significant challenge to livelihoods (e.g., death of livestock, acute food shortage).
- Stress is a slow-onset or chronic occurrence of recurrent droughts resulting in a significant challenge to livelihood (e.g., psychological distress, trauma).
- Vulnerability factors: factors (e.g., lack of skill, value placed on livestock, mind set) that make people, infrastructure, and institutions in these communities vulnerable to recurrent droughts.

### 2.7. Trustworthiness 

Various quality control measures were implemented to ensure the trustworthiness of the findings. Data collectors and fieldwork supervisors were recruited from multidisciplinary backgrounds and they were trained well. The appropriateness and relevance of the data collection tool was ensured through expert reviews and pre-testing. The field team was holding debriefing session each day during the entire field work. Facilitators’ and note takers’ impressions were documented for each data source. Data from FGDs and KIIs were triangulated to ensure credibility. In order to get feedback on whether the interpretations and the dimensions emerged from the findings make sense, the study results were presented at three consultative workshops that representatives of the target communities attended, and provided feedbacks.

### 2.8. Ethical Considerations 

The study was approved by the Institutional Review Board of Jimma University (Ref. No.: HRPGC/2015/2013), and permission secured from all relevant offices. Informed verbal consent was obtained from each participant.

## 3. Results

### 3.1. Resilience Dimensions

**Figure 1:**
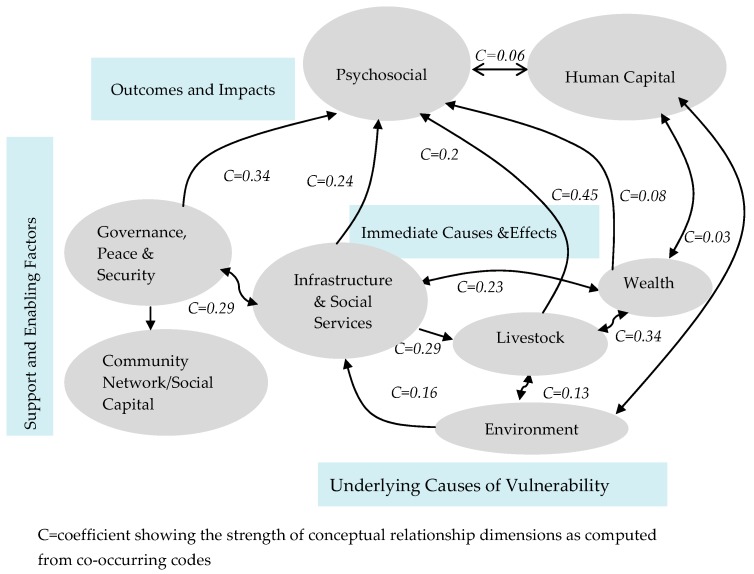
Resilience dimension framework-relationships between resilience dimensions in the analysis of recurrent droughts in pastoralist communities of Borana Zone, Southern Ethiopia.

The findings are organized in a conceptual framework named Resilience Dimensions Framework (RDF). This framework was developed following coding and clustering of codes on aspect of resilience towards impacts of recurrent droughts (Figure 1). The following section gives a description of each of the emerged resilience dimensions with respect to adaptive capacities, vulnerability factors and cause-effect chains.

### 3.2. Environment

The result shows that environment was the most frequently discussed resilience dimension as related to recurrent droughts; and it contains several resilience elements that include environmental rehabilitation and protections; range land and natural resource management; soil protections; afforestation; water resource management; and climate change issues. Many respondents mentioned that natural factors such as environmental variability and climate change are actually driving recurrent droughts in the area. They believed that arid nature of the environment accompanied by bad weather condition, absence or diminishing forests and perennial rivers, expansion of invasive plants and erratic rainfall play major role in recurrence of droughts. One male FGD discussant said,

“In my opinion, the causes of drought can be the dry nature of our land which does not bring appropriate wind carrying rainfall.”

(FGD, male)

On the other hand, several respondents also mentioned that human activities such as deforestations, bush encroachment, charcoal productions, unwise use of resources, overgrazing, population growth, and large herd size contributed to environmental degradation. Many respondents agreed that environmental management and protections must be emphasized.

“Our land is arid with dry weather condition. So, we do not have forest or trees. Therefore, we need to plant trees to protect the environment.”

(FGD, male)

### 3.3. Wealth

The analysis indicated that wealth was found to be one of the strongest dimensions to cope with, recover from and adapt to the impacts of recurrent droughts. It was closely and strongly linked to livestock, infrastructures and psychosocial wellbeing (Figure 1). This dimension encompasses a wide range of resilience elements such as role of alternative livelihood opportunities (e.g., gum production and agricultural farming), adopting values and norms that can accept alternative livelihood. This dimension also describes financial assets (e.g., saving money, access to saving and credit services) and non-financial assets (e.g., building a house in a town), as well as asset redistribution.

Moreover, it is related to food availability and access to adequate and safe food and non-food items necessary for survival. Many participants generally emphasized that poor livelihood diversification (i.e., sole dependency on livestock) practices as major vulnerability factors that affect community’s wealth conditions. One key informant said:

“People use cattle as their food and cloth, to send their children to school and as their means of generating household income. Drought has impoverished most of the households and degraded their capacity, worsening their Vulnerability.”

(KII, Borana)

Moreover, several participants consistently mentioned that illiteracy, combined with restrictive cultural norms and values would led to poor saving habit and unsupported attitudes towards participating in alternative livelihood activities:

“We are less familiar to participate in other job activities.”

(FGD, male)

Some respondents mentioned that there were households who were trying various livelihood strategies such as crop production, building or buying houses in urban areas to rent and get additional income; fattening oxen and saving money in bank. traditional asset redistribution (e.g., livestock restocking (*Busa Gonofa*) and loaning milk cows (*Dabare*) from wealthy households to the poor. One key informant said:

“People have started selling their cattle and save the money in the Bank.”

(KII, Borana)

A few participants said that some households earned income from forest products, from which they could produce gum to make money that would help them to cope with the effects of drought.

There was a general agreement that these adaptive strategies were insufficient to build the resilience of the local communities and hence, many participants of FGDs and KIIs suggested various interventions that would boost households wealth status such as expanding alternative livelihood opportunities through skill development and encouraging participation in crop farming, initiating irrigation technologies; ensuring access to financial services and promoting saving and credit services, small group microfinance, entrepreneurship, business development (with initial capital support), fattening oxen; engaging in trade/business (e.g., opening a shop); engaging in gum production; financial and non-financial asset development (e.g., building a house in town and renting it); reducing mobility and adopting a settled lifestyle.

### 3.4. Livestock Management

This resilience dimension revolves around factors that determine people’s adaptive and coping strategies in managing their major source of livelihood such as role of proper livestock management skills, increasing livestock productivity, herd size control and diversifications, and introducing improved and drought resistant livestock species. Further, it addresses some important livestock services including access to drinking water, animal health care, fodder/forage production and management, proper grazing land management; role of livestock insurance, and rehabilitation services to build livestock related resilience.

Many participants mentioned that cultural norms and values that favored large herd size; poor herd diversity; lack of forage or fodder; scarcity of water and veterinary services; poor livestock management skill and lack of market linkage for their livestock products were key vulnerability factors:

“We value to have as many cattle as possible.”

(FGD, female)

A key informant also said:

“In Borana community people like to rear cattle more for social value than for selling and feeding themselves.”

(KII, village level)

It was mentioned that some people are increasingly establishing private grazing land which restrict access by others:

“In the previous times, there were *Kalos* (area enclosures) commonly used by the whole community. But these days, some people fence their own *Kalo* and do not allow others to use it.”

(KII, Borana)

The result shows that communities were using various indigenous adaptive strategies to manage the impacts of recurrent droughts on their livestock though they were not sufficient enough to help them. The major ones included migrating to safer places and temporary self-resettlement, segregating herds to give priority to calves and lactating cows, enclosing grazing land (*Kalo*), as well as purchasing and preserving fodder. A key informant said:

“One way is dividing the animals into different groups. We separate the weaker ones from the stronger ones during grazing. The breast feeding and non-breast feeding cows are also kept separately.”

(KII, village level)

Some respondents also mentioned that some households sell their cattle before the onset of drought and save the money in a bank to use it during drought or to buy cattle when the drought ended:

“Households who sold their cattle and purchased grains for food and those who deposited money in bank are better to withstand the effect of drought.”

(FGD, male)

In order to promote community’s resilience on livestock, many participants suggested several interventions like acquiring or breeding drought-resistant cattle, controlling herd size and diversification, developing community skill on livestock management, grazing methods, digging ponds and large reservoirs for water preservation and rehabilitating existing water sources through communal efforts; promoting and increasing access to animal insurance; proving animal health services and promoting saving habit by selling some cattle:

“If one has ten cattle he/she has to sell two or three of them and deposit the money in Bank to use for purchasing fodder. This method can be effective if accompanied by supports or aid from government.”

(FGD, informal leader)

“Diversifying our livestock by replacing or increasing the number of Camels and goats is important. As compared to cattle, they better resist drought.”

(FGD, female)

### 3.5. Infrastructure and Social Services

This dimension emphasizes the role of access to essential infrastructures and social services such as water supply, basic human and animal health services, appropriate and effective early warning system, timely information on disaster preparedness and weather conditions; access to educational facilities, market services and information, and to road (to get connected to market places and resources in other areas). Many FGDs and interview participants explained that limited access to these basic infrastructures were crucial challenges:

“This land has been lagging behind in education. There is no school around. Our children have to travel long distance if they have to get education. It is far away from here and that is also very poor one. Besides, there is problem of water. There are many cattle and they all need water, but it is insufficient. Sometimes, you fail to find any water for drinking.”

(FGD, male)

Lack of or limited animal health care service also remains critical challenge to keep their livestock healthy and productive:

“Our cows usually abort which could be due to lack of vaccination. Drought has occurred again and again devastating our cattle.”

(FGD, female)

Another FGD participant added:

“Gradually they (cattle) lose their nails, becoming weak, skinny and emaciated due to lack of health care and drugs.”

(FGD, female)

It was also reported that lack of access to markets prevents the community members from selling their livestock products at good prices. Respondents mentioned that community members were forced to sell their livestock at lower prices but purchase cereals at higher prices. Some mentioned that the government tried to fix market price but that intervention rather aggravated the price inflation. A key informant said:

“Drought in one area affects the situation in another area by influencing prices in the local market; that is it increases grain prices but decreases livestock prices.”

Another key informant said:

“The government fixed price for each item but nobody wanted to sell in that price!”

### 3.6. Enabling and Support Oriented Resilience Dimensions

Two resilience dimensions (social capital/community network as well as governance, peace and security) emerged as supportive to other resilience dimensions. According to the data, the study communities had an indigenous social security system, either as compulsory or on voluntary basis, which takes the form of asset redistribution or restocking drought affected families (*Busa Gonofa*); providing milk cows to drought affected households on a temporary basis (*Dabare*); collecting and redistributing milk to poor households (*Busa-konki*); and a traditional mutual support system in which community members get together to help a neighbor with major tasks (*Debo*). Most participants believed that these traditional support systems were crucial social capital in Borana communities in rehabilitating and capacitating drought affected families. A key informant said:

“They (community) always support each other through traditional mechanism; supporting one another through *Busa Gonofa* based on kinship or clans. Poor persons get support.”

One FGD participant also confirmed that:

“A person who survived with one cow should provide milk to other family; one who is left with two cows must give one cow to his brother or the other Borana. We call this method of support *Busa Gonofa*. If you refuse *Busa Gonofa*, it is crime in our laws and you face punishment.”

(FGD, female)

However, many participants ascertained that these indigenous social support schemes were weakened, and are not properly functioning in recent years due to various reasons: First, it was mentioned that households’ ability to contribute has been degraded. Some explained this as, “No one is better than any other person”. It was also mentioned that frequent stresses had made people unwilling to share resources:

“The number of cattle each household own has decreased as a result of drought and shortage of fodder. This has weakened our *Busa Gonofa*.”

(FGD, female)

Some participants also stated that external aid encouraged dependency and eroded the indigenous tradition of sharing resource. There was also a report that formal governance structure negatively affected the indigenous governance system thereby weakening the indigenous social scheme:

“The government has a role in weakening our traditional social network which we have been using for centuries. Because the government gives us aid in different forms: money or materials. These aids affect our capacities because we are depending on these aids and looking to get more of it.”

(FGD, female)

Many FGDs participants and some key informants explicitly discussed that lack of good governance, peace and security in these pastoralist communities was one of the greatest resilience challenges. In many FGDs (9 of 12 FGDs) and key informant interviews (27 of 36 interviews), it was frequently mentioned that drought often induces violent conflicts between ethnic groups or adjacent communities due to fighting over scare resources, particularly over water sources, “*Ela*” and grazing lands. One FGD participant sensitively explained this issue as:

“Always we have serious conflict with Garri (ethnic group) over our land, ponds and *Elas*. This has become a serious problem to us. We consider *Ela* is wife for Borana. The *Elas* have rules and procedures governing their use, which we call *Konfi* in Borana culture. It is not possible to take away one’s wife and give to someone else. Doing this leads to violent conflicts.”

(FGD, female)

According to them, the fact that migration was the major coping mechanism during drought event, it often led to hostile competition over scarce resources. A key informant said:

“People quarrel over water and grass when drought occurs. For instance, there are people from region five (Ethiopian Somali) on this side. People rob each other at this time.”

(KII, Borana)

Furthermore, many respondents argued that the formations of new boarders and delimitations by the government contributed to instability in the area. One FGD participant explained this as follows:

“Previously the land of this people had no boundary. Our cattle did not run shortage of grasses; there were trees, and enough wells. Now, this land has been restricted. It was taken from us and given to others in plots of five! People were made to evacuate from the land.”

(FGD, male)

A key informant from one of the study districts also stated:

“Always there is conflict around boarder areas with region five (Ethiopian Somali). These boarder areas are suitable for grazing. When we move to these areas to search for pasture, the Somali attack us within two to three days of our arrival and as a result development activities are being discontinued and people are displaced from their residence. The source of this conflict is inappropriately drawn boundaries and as a result, it has been attracting inter-ethnic conflicts.”

(KII, Borana)

The result shows that, under the umbrella of *Gada* system, the community had an indigenous conflict management and resolution practices to maintain peace and security. However, these traditional means of conflict resolution are less effective these days and many participants suggested strengthening indigenous conflict management and resolution mechanisms while addressing disparities in basic infrastructures and social services and promoting good governance. Some FGD participants strongly commented that resolving boarder related issues are prerequisite to building resilience efforts. One participant said:

“If we do not get back our land, we cannot think about other activities like farming, trade or development. The government is always organizing various meetings and telling us about development; teaches us about involvement in business oriented activities. But, we do not have land on which we can carry out development. Let me tell you the truth! We are always being concerned about the issue of our ponds and *Elas* than drought. In this regard, our voice and rights are disregarded.”

(FGD, male)

### 3.7. Outcomes and Impacts Oriented Resilience Dimensions

Several FGD participants and key informants explained that community members often live in stressful situation. They used different expressions to describe the psychological experience of community members such as depression, distress, anxiety, frustration, fear, and hopelessness:

“The main reason why we fear is that the performance of pasture and herbaceous plants for our animals are not as good as before.”

(FGD, informal leader)

“The rain has completely stopped and even now the signs we are observing shows that drought is approaching. So, we are very scared.”

(KII, community level)

“Households who lose all of their cattle become psychologically abnormal or face mental problem. Some households may lose up to 300 cattle at once and this makes them mentally ill.”

No research participant mentioned any intervention in place to help these community members with psychological problems. It was frequently mentioned that people mostly relied on praying to God to get divine support:

“We believe that the “*Waaqa*” (God) is our supporter during such terrible time. We know that when such challenging things happen, God would interfere.”

(FGD, informal leader)

Similarly, the resilience analysis showed that human capital is one of the cornerstones in resilience to drought effect and is emphasized on the role of community’s knowledge and skill, capacity, education and training, leadership and school support services to enable people to manage the risks of drought. The analysis revealed that recurrent droughts eventually lowers the stock of human capital as it distracts educational infrastructures and supporting facilities; limits access to school and causes huge school dropouts. A 36 years old male FGD participant said:

“Families move away from their dwelling place because of drought, and children are forced to drop out of schooling.”

Another male FGD participant also said:

“Drought causes schools and health centers to be closed, and people are forced to move in search of food and pasture.”

It was frequently mentioned that lack of awareness or education and life skills limited people’s ability and knowledge to manage their resources and living conditions, increasing their vulnerability to the impacts of recurrent droughts:

“The basic reason why we fail to cope with drought is lack of education.”

(FGD, male)

Several participants suggested that empowering community members, improving their skills and knowledge through various methods such as training, modeling, awareness creation and attitudinal change are essential interventions:

“Maximizing the capacity of the community by giving different trainings is important. The capacity may be in terms of improving economic capacity or knowledge or skill.”

(KII, Borana)

Figure 1 shows the resilience framework that emerged from the analyzed qualitative data. The framework contains eight resilience dimensions for these specific pastoralist communities. These resilience dimensions are organized in relation to each other taking into account the extent of thematic and conceptual proximity and linkage among them. The dimension-by-dimension co-occurrence frequencies and coefficients from ATLAS. ti 7.1.4 determined how closely the dimensions were related. Moreover, the qualitative facts influenced the leveling of the dimensions and the state of the links between them. Accordingly, psychosocial and human capital dimensions were mostly discussed as the eventual outcomes and impacts of recurrent droughts, and thus, are placed at the top of the framework. Environment was mostly described as underlying cause of vulnerability pertaining to recurrent droughts and goes to the bottom of the framework. Wealth, livestock as well as infrastructures and social services resilience dimensions co-occurred a lot and were mostly related to the immediate causes and effects of recurrent droughts and thus, kept at the center of the framework. Community network and governance, peace and security dimensions were mostly related to supporting and enabling factors for a variety of adaptive and coping strategies in livestock, wealth and infrastructures/social service dimensions. This framework was developed through interactive process among investigators who deeply immersed themselves in the dataset and was subjected to critics and continuous feedbacks from experts, stakeholders and representatives of the study communities. It was presented, commented and revised on a series of three consultative workshops in Jimma (April 2014)-Ethiopia, Kampala (May 2014)-Uganda and Addis Ababa (July, 2014)-Ethiopia and found relevant and appropriate to enable pastoralist communities and system to be resilient to the impacts of recurrent droughts.

## 4. Discussion 

This qualitative study explored resilience dimensions and adaptive strategies towards the impacts of recurrent droughts among pastoralist communities of Borana in Southern Ethiopia. In order to build community resilience system, it is vital to understand what makes people and systems resilient to the impacts of recurrent droughts in a specific context. Drought risk reduction and resilience strengthening attempt requires context driven strategies that are devised through in-depth understanding of local perspectives and indigenous drought response, practice and knowledge. In most circumstances, drivers of drought and vulnerability factors are quite local with specific characteristics that must be understood for the determination of appropriate measures to reduce risk and improve resilience [28]. Nevertheless, most resilience building programs are guided by a broader global, regional, or national level framework [29] which basically lack essential elements of sensitivity and compatibility to the local needs, priorities and aspirations of the target community. Such top down approach often fails to recognize the important role of communities, and ignore the potential of local knowledge, resources and capacities, and they may even saliently increase people’s vulnerability to shocks and stress [19,20,29]. Given that the nature and characteristics of vulnerability factors and stresses are variable from place to place, resilience interventions that drive from contextually irrelevant theoretical framework is fundamentally ineffective [19,20,21,22]. We argue here that the resilience of people and systems shall be strengthened by designing and implementing innovative interventions identified by a scientific, data-driven and evidence-based resilience framework. The present study inductively constructed a resilience framework; addressing the gaps in the science of resilience which often guided by underdeveloped understanding of the dynamics and causal pathways of resilience in a specific context. Thus, the emergent framework is useful to design and implement contextual and locally appropriate resilience interventions to boost pastoralist capacity to withstand impact of drought.

The present resilience framework consists of eight dimensions- two outcome oriented dimensions (psychosocial distress and human capital), underlying cause of vulnerability (environment), immediate cause and effects of vulnerability (wealth, livestock, infrastructures and social services) and two supporting and enabling factors dimensions (community network and governance and peace and security). The framework depicts the relationship among these resilience dimensions in the context of recurrent droughts and emphasizes on understanding what makes people and systems capable to withstand or adapt to impacts of recurrent droughts in such a way that they make the communities less vulnerable to future risks and vulnerability.

In this framework, environment has been identified as an underlying cause of vulnerability and constitutes drivers of adaptive capacity as well. Degradation of range land is a major cause of underlying vulnerability suggesting that rangeland management is an important foundation upon which community’s resilience capacity could be built. Resilience interventions that address environmental aspect, combined with climate sensitive approaches, enable the communities to better manage their environment and cope with the impacts of recurrent droughts [30]. In this domain of resilience, it is vital to address negative coping mechanisms such as deforestation and imprudent use of natural resources, poor grazing land management, poor community’s skills and knowledge on environmental management and protection. The livelihood of these pastoralist communities was closely and strongly linked with traditional water sources, *Elas*. However, the present study indicated that, due to decreased and erratic rainfall combined with mismanagement and over exploitation, the functionality of these water sources has diminished which was also reported earlier [31].

Livestock management is among important resilience elements that emerged from the current data. Livestock production was almost the sole means of livelihood in the study area. People rely on livestock and its products for food; serving as basic means of household income to purchase basic household necessities and to send their children to school. Thus, in the face of this heavy dependence on livestock production, drought that affects livestock productivity at any level has an immense negative impact on household’s entire livelihood system. Even though livestock is at the heart of the pastoralist livelihood, many constraining factors limit their economic contribution to household wellbeing including, scarcity of drinking water, shortage of pastures and prevalence of animal diseases. In addition, the existing livestock is less diversified, and combined with communities’ limited skill and knowledge on livestock management and grazing technique, it created burden over grazing land and worsen the problem. By diversifying their livestock, pastoralists can generate a wider variety of livestock products and make better use of the available forage in different seasons, even in times of crisis. On top of that, lack of access to market and market information remain key constraint to effective livestock production in this community; adversely affected pastoralist resilience capacity which was also noted in earlier report [32]. This is because the demand for livestock products often falls during drought seasons which forces the pastoralists to sell their cattle at lower prices but purchase grains and other necessities at higher prices.

Of course, communities were using various coping strategies though some of these coping strategies had negative impacts on long term resilience capacities. During drought, the pastoralists migrate to other places in search of pasture and water. This mobility enables them to take advantage of pasture resources that are only seasonally accessible. However, mobility has been increasingly restricted due to new boarder formations and demarcations which was also reported in previous study [33]. Cattle segregation was another important coping strategy being used by these pastoralist communities. Such strategy is essential to reduce competition among herds for pasture/forage and water resources and it optimizes pasture use [33]. Resilience interventions are required to strengthen or promote this practice on large scale. The cultural values and norms placed on livestock (e.g., keeping large number of cattle) is a serious constraint to herd size control and it is necessary to design effective resilience strategy that influence the mindset of the pastoralist communities.

In the emerged resilience framework, household wealth status appeared as a cornerstone because it contains essential resilience elements such as access to alternative livelihood opportunities and diversifying financial and non-financial assets. Evidence has also supported that diverse livelihood opportunities are closely linked to improved resilience capacities in pastoralist communities [12,34,35]. Therefore, any resilience interventions in pastoralist communities need to expand various forms of livelihood opportunities such as supporting income-generating activities (e.g., processing and selling of livestock products, gum productions, expansion of trade) as a way to enhance household socio-economic status. On small scale, there was an attempt to diversify livelihood among some households and further resilience building efforts could be built on community’s own initiated strategies. However, consistent with previous reports [36,37] these communities engaged in harmful alternative livelihood mechanisms such as charcoal production which further deteriorate their resilience capacities in the long term, and this merits special attention. In this regard, providing supports and tailored resilience interventions such as technical and vocational skills and business training, facilitating access to microfinance services, saving and loan groups are appropriate to strengthen community resilience system. In fact, given the existence of deep rooted and widespread cultural values and norms associated with livestock production, there could be resistance to adopting alternative livelihood opportunities, calling for addressing attitudinal and cultural factors along with expanding livelihood opportunities.

Another important and cross cutting resilience element for pastoralist communities is related to infrastructures and social services. In the present framework, underdeveloped physical infrastructures and social services are strongly linked with weak resilience. Poor infrastructures and inequitable distribution of resources and facilities, particularly water resources, often trigger violent conflicts among pastoralists due to competition over this limited resource. This prevents access to existing infrastructures and blocks proper utilization of resource. Similarly, limited access to road, poor market opportunity and poor access to metrological information and early warning system limit communities’ adaptive capacities. This has also been indicated in an earlier study [37]. Thus, ensuring equitable access to basic infrastructures and services, mainly water resources; integration of markets and increasing interconnectivity; establishing effective and timely drought warning and metrological information must be a top priority for pastoralist communities.

The study also identified that the Borana pastoralist communities have strong traditional institutions and scheme that play a vital role in periods of stress and shocks, including recurrent droughts. One such social indigenous scheme is *Busa Gonofa* defined as clan based asset redistribution and restocking drought affected families. These communities had also several other indigenous social support mechanisms such as *Dabare*, *Busa-konki* and *Dabo* which are crucial in a variety of positive adaptive and coping strategies. Through these indigenous mechanisms, communities were able to manage risks, and promote collective actions for mutual social security and peace. Unfortunately, these important social capitals have been degraded due to increasing demands, decreasing capacities to contribute and influence of modern government structures and external aids; leaving affected families without care and support. Some earlier studies also reported significant deterioration of this system [38,39]. Thus, it is highly important to strengthen and revitalize these indigenous, institutions and relationship as part of resilience building activities.

Another important resilience dimension that provides an essential enabling environment for adaptive and coping strategies is sustainable peace and stability. Pastoralist communities generally rely on mobility as major coping strategy. However, this makes them more vulnerable to violent conflicts over scarce resources which could result in many consequences on pastoralist life such as limiting economic activities, destruction of resources, and facilities, and also can cut off their access to key resources and block them from important markets. The fact that peace and security has strong linkage with many other resilience dimensions particularly psychosocial, wealth, human capital and infrastructure proves that peace building and stability must be a core component of any resilience building activities in pastoralist communities. Some earlier studies also documented that peace and security is vital to build resilience capacities and coping strategies in pastoralist communities [34,37,40,41,42]. It is essential to promote or strengthen indigenous and customary systems of conflict management and resolution so that pastoralists are better able to negotiate resource during drought events. In this case, promoting the *Gada* System, an Oromo indigenous socio-political system governing all community affairs [43], has been mentioned by many respondents as vital and hence, resilience interventions efforts need to integrate indigenous peace building scheme However, many risk management and resilience interventions often do not stress the role of focusing on peace and security, and social capital as integral part of resilience building efforts [29,44]. In conflict context, peace building that aims to improve healthy relationships among groups can potentially help households’ better cope with shocks and stress [45].

As related to gradual onset and long term effects of recurrent droughts, these pastoralists live with stress and anxiety. This mounting psychosocial stress and sense of hopelessness could affect societal relationships and productivity since affected individuals become increasingly passive; lack a broader and optimistic vision of the future. This significantly affects resilience capacity which is also noted elsewhere [46,47,48]. Unfortunately, these pastoralists lack any acceptable coping strategy to improve their psychological wellbeing and it is therefore important to design effective support mechanism to deal with psychosocial stress and trauma and promote optimistic future vision.

Recurrent droughts gradually affect human capital in pastoralist communities as educational achievements are seriously impacted due to disruption of educational infrastructures and instability. Furthermore, drought demands a higher workforce to care for livestock and as a result families are forced to stop sending their children to schools. Conversely, poorly developed human capital is less capable of choosing positive adaptive strategies which is also reported elsewhere [49]. Thus, community oriented and tailored awareness creation program, and education that incorporate endogenous knowledge and relevant content for skills, attitudinal and knowledge based trainings to human capital for pastoralists are very critical.

The major strength of the study was the use of inductive approach to devise contextually relevant and data driven resilience framework which is a critical step in building sustainable resilience towards impact of recurrent drought. It provided insight on ways to understand, conceptualize and develop contextually relevant and measurable resilience framework towards strengthening capacity of pastoralists to resist and withstand impact of recurrent drought. The conceptual framework can serve as a specific and focused resilience assessment tool making easier assessing effectiveness of resilience interventions which is one of the critical gaps in resilience programing [21,44]. We anticipate that the present study contributes to current growing body of literature around resilience and vulnerability, particularly in the area of drought and pastoralism. The framework and the methodology used in the present study can be replicated and adapted in similar context outside Ethiopia.

This study has some limitations. As the study participants were adults, the views of young people were not included and this might limit adaptability of the emergent conceptual framework. The framework is developed on the basis of the perceptions and perspectives of communities on resilience and that it should be acknowledged in interpreting the findings. In fact, resilience work should be shaped by the perspectives, realities, and priorities of the target communities which ensure communities’ receptivity and acceptability of the proposed resilience interventions. In addition, even though views of women were adequately captured in the study, the framework did not differently illustrate role of gender in response to drought. Some evidence indicated that women often experience stress and hardship differently; and the couples who shared the burden through mutual decision making exhibited better resilience [50]. The framework did not differently treat people with disability; rather it suggests resilience building at community and household levels, which could benefit community members as a whole. Additionally, the study did not cover large geographical areas which might limit the scope of application of the resilience framework.

## 5. Conclusions 

The resilience capacities and indigenous adaptive strategies of the pastoralist communities have been eroded by recurrent droughts and the concomitant effects. The study depicted that resilience is multi-dimensional having eight interrelated components with complex interactions and relationship informing that communities’ and system may be enhanced by acting at different resilience pathways as potential entry points for intervention. The analysis indicated that resilience does not exist in a single resilience metric; instead, the entire framework together, interacting with one another constitutes resilience. The framework is context specific and it is very useful to guide resilience programming to enable the communities and the system to cope up; adapt to, and recover from the impacts of recurrent droughts. Basically building resilience against impacts of recurrent droughts requires interventions that strengthen the eight components together and at multiple levels (individual, households, communities and system). However, resilience elements which are kept in the center of the framework (wealth, livestock and infrastructures and social services) would have stronger influence on community and system resilience. Yet, any resilience building intervention needs to consider the enabling and support related resilience elements which include community networks as well as governance, peace and security. While the framework provides useful guidance to understand, initiate and design context specific resilience programming, it is also important to test the applicability, conceptual and statistical relationship among the resilience elements through appropriate sample size and survey method. The study also found that the top down and crises management approach remains one of the key resilience; saliently affected long term capacity of the local community, Thus, future resilience initiatives must reduce pastoralists’ dependence on external support; rather contribute to development of locally appropriate innovative solutions that would complement long term resilience building in pastoralist communities.
